# The implementation and utility of patient screening logs in a multicentre randomised controlled oncology trial

**DOI:** 10.1186/s13063-020-04559-w

**Published:** 2020-07-08

**Authors:** Rebecca Lewis, Rachel Todd, Michelle Newton, Robert J. Jones, Caroline Wilson, Jenny L. Donovan, Richard T. Bryan, Alison Birtle, Emma Hall, on behalf of the POUT Trial Management Group

**Affiliations:** 1 Clinical Trials and Statistics Unit at the Institute of Cancer Research (ICR-CTSU), London, SM2 5NG UK; 2 Institute of Cancer Sciences, Beatson West of Scotland Cancer Centre, University of Glasgow, Glasgow, UK; 3 Population Health Sciences, Bristol Medical School, Bristol, UK; 4 Institute of Cancer & Genomic Sciences, University of Birmingham, Birmingham, UK; 5 Royal Preston Hospital, Lancashire Teaching Hospitals NHS Foundation Trust, Preston, UK

**Keywords:** Patient screening logs, Trial recruitment, Rare cancer

## Abstract

**Background:**

The utility of patient screening logs and their impact on improving trial recruitment rates are unclear. We conducted a retrospective exploratory analysis of screening data collected within a multicentre randomised controlled trial investigating chemotherapy for upper tract urothelial carcinoma.

**Methods:**

Participating centres maintained a record of patients meeting basic screening criteria stipulated in the trial protocol, submitting logs regularly to the clinical trial coordinating centre (CTC). Sites recorded the number of patients ineligible, not approached, declined and randomised. The CTC monitored proportions of eligible patients, approach rate (proportion of eligible patients approached) and acceptance rate (proportion recruited of those approached). Data were retrospectively analysed to identify patterns of screening activity and correlation with recruitment.

**Results:**

Data were collected between May 2012 and August 2016, during which time 71 sites were activated—a recruitment period of 2768 centre months. A total of 1138 patients were reported on screening logs, with 2300 requests for logs sent by the CTC and 47% of expected logs received. A total of 758 patients were reported as ineligible, 36 eligible patients were not approached and 207 declined trial participation. The approach rate was 91% (344/380), and the acceptance rate was 40% (137/344); these rates remained consistent throughout the data collection. The main reason patients provided for declining (99/207, 48%) was not wanting to receive chemotherapy. There was a moderately strong correlation (*r* = 0.47) between the number reported on screening logs and the number recruited per site. Considerable variation in data between centres was observed, and 54/191 trial participants (28%) enrolled during this period were not reported on logs.

**Conclusions:**

Central collection of screening logs can identify reasons for patients declining trial participation and help monitor trial activity at sites; however, obtaining complete data can be challenging. There was a correlation between the number of patients reported on logs and recruitment; however, this was likely confounded by sites’ available patient population. The use of screening logs may not be appropriate for all trials, and their use should be carefully considered in relation to the associated workload. No evidence was found that central collection of screening logs improved recruitment rates in this study, and their continued use warrants further investigation.

**Trial registration:**

ISRCTN98387754. Registered on 31 January 2012

## Background

The POUT multicentre randomised controlled trial (a phase III randomised trial of Peri-Operative chemotherapy versus sUrveillance in upper Tract urothelial cancer, CRUK/11/027) investigates the role of adjuvant systemic chemotherapy in patients with upper tract urothelial carcinoma (UTUC) [1]. UTUC affects the ureter and renal pelvis and is a rare disease [2], with an approximate annual incidence of 1–2 cases per 100,000 people [3]. Standard treatment comprises surgery to remove the affected kidney and ureter (nephroureterectomy), followed by surveillance for recurrence. Incidence of recurrence is high, with disease returning within 5 years of the initial surgery in 30–50% of patients with localised disease [4]. There are few data available regarding optimal adjuvant treatment strategies following surgery, and uncertainty exists regarding the value of chemotherapy, with no international consensus [5].

**Fig. 1:**
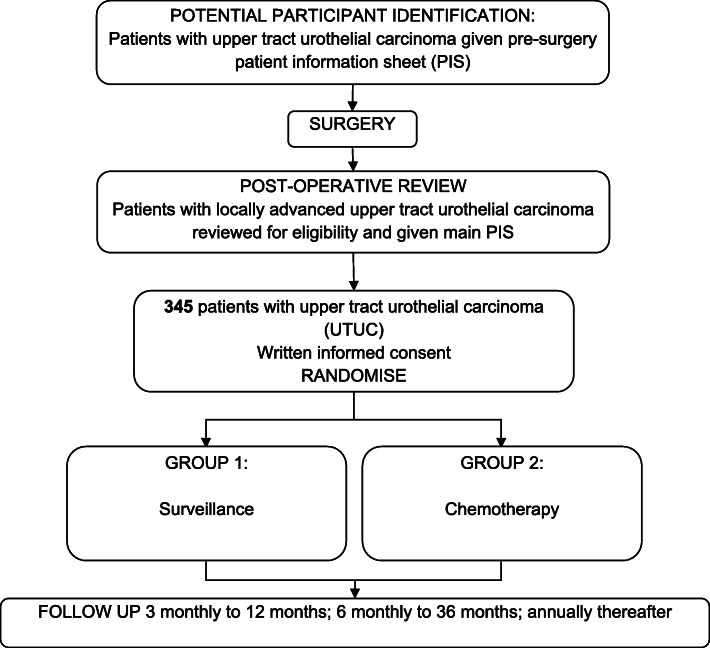
POUT trial schema

The POUT trial aims to establish whether UTUC is sensitive to platinum-based chemotherapy. The primary outcome measure is disease-free survival (time from randomisation to cancer recurrence or death), with secondary outcome measures including safety and quality of life. Following surgery, participants were randomly allocated to either adjuvant chemotherapy or surveillance, with treatment according to local practice if recurrence occurred (Fig. 1). Participants were identified by their urologist or oncologist, recruited from secondary care hospitals across the United Kingdom (UK), and could be approached about participation either before or after surgery.

It is well established that recruitment to clinical trials can be challenging, with surveys of clinical trial coordinating centres (CTCs) in the UK identifying the improvement of recruitment rates as a top priority [6, 7]. It was anticipated that recruitment may be particularly difficult in the POUT trial due to the difference between the treatment strategies being investigated [8, 9]; the investigators thought potential participants may be disinclined to join a trial in which they could be allocated to a group which did not receive immediate treatment, as has been observed in other studies [10, 11]. In order to raise awareness of the trial at an early stage in the patient pathway, a brief pre-surgery patient information sheet was prepared to introduce the trial to patients prior to nephroureterectomy. This was in addition to the main patient information sheet, given to all those approached following surgery as part of the written informed consent process. A qualitative recruitment study was initiated to investigate recruitment activity at sites in depth [12]. Sites were also provided with a screening log with the intention of tracking potential participants and helping the CTC to centrally monitor recruitment activity at sites.

Screening logs were implemented as a result of the investigators’ prior experience, including within the SPARE trial (a feasibility study comparing treatment modalities in muscle-invasive bladder cancer) [13], as a tool to centrally monitor and support recruitment activity at sites. Screening log data collected in the SPARE trial indicated that recruitment to a large-scale phase III trial was not feasible due to a lack of eligible patients and the trial therefore closed early [8]. The use of screening logs is recommended by the UK Medical Research Council’s Hubs for Trials Methodology Research Recruitment Working Group in their advice on optimisation of recruitment [14]. They suggest screening logs act to raise awareness of the trial, ensure all potential recruits are reviewed, and enable central review of eligibility criteria.

Previous studies reporting use of screening logs have used the information gathered to justify expansion to additional sites and revision of eligibility criteria, and to monitor recruitment rates [15–17]. One study found that sites submitting fewer than 50% of expected logs for two stroke trials achieved half the monthly recruitment rate of those submitting over 50% [18]. This was supported by similar findings in another study which demonstrated that sites with the highest recruitment screened the most patients per month and recruited a higher proportion of patients than lower-recruiting sites [19].

It has, however, been noted that there is no standard definition of a ‘screened patient’ [19]; therefore, data can be difficult to generalise. This represents a challenge when trying to compare data between studies and raises questions about the validity of including screening data in randomised controlled trial publications, as recommended in the latest version of the CONSORT Statement [20]. The recent SEAR (Screened, Eligible, Approached, Randomised) publication [21] suggests a standardised framework for collecting screening data similar to that used in the POUT trial and, if adopted, may facilitate generalisability between trials in the future.

The aim of this exploratory retrospective analysis is to investigate the utility of screening logs within the POUT trial, assess the impact of one of the changes to the trial documentation made midway through the screening period, investigate correlations between reported screening activity and actual recruitment and identify potential topics for future prospective studies.

## Methods

**Fig. 2:**
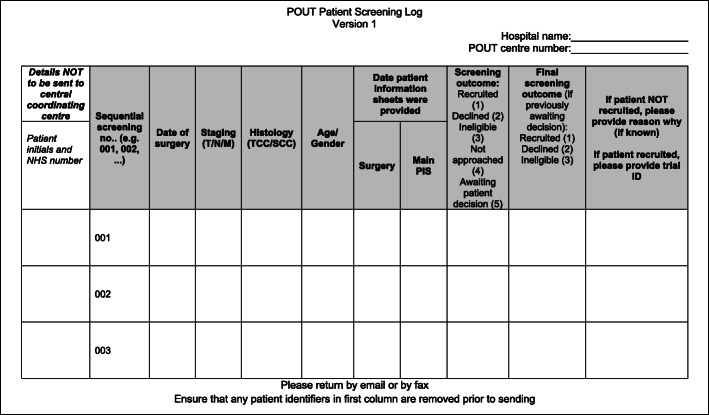
Screening log template provided to participating sites

Research teams at participating NHS hospitals were provided with a screening log (Fig. 2) based on the CTC’s standard template, and screening criteria were defined in the trial protocol. Sites conducting surgery were asked to record all patients receiving nephroureterectomy for suspected UTUC. Sites to which patients were referred following surgery were asked to record all patients fulfilling the core eligibility criteria, i.e. diagnosis of locally advanced, non-metastatic UTUC. Data requested included basic eligibility information and details of the recruitment process. The aim was to capture information about all patients fulfilling the screening criteria, with date of surgery, histological details, dates both patient information sheets were provided and the recruitment status (ineligible, not approached, declined, randomised, pending or awaiting patient decision). If patients were happy to disclose the reason they declined participation, this was also recorded. Each patient was allocated a sequential screening number, and the status was updated by the site as required until a final outcome was reached (ineligible, not approached, declined, randomised). No patient identifiers were collected centrally; therefore, patient consent was not required.

The log was provided to centres as a Microsoft Excel spreadsheet, and it was recommended that it was used as a local tool to track potentially eligible patients from before surgery to the final outcome. Sites were asked to submit the logs to the CTC monthly. The CTC also sent regular reminder emails to request logs from all sites which had been open for at least 1 month.

Upon receipt, logs were reviewed for any discrepancies in eligibility criteria. If any patients appeared to have been incorrectly deemed ineligible, this was raised with the site in real time with the aim of ensuring no eligible patients were overlooked. Sites were also regularly reminded to report all recruited patients on the logs if any had been omitted.

Each log received from sites was entered into a central MS Excel spreadsheet at the CTC, and data were cleaned to remove any patients reported in error, for example, patients who had not had surgery or those who did not have UTUC. Incorrect reporting of patients was notified to sites for training purposes.

Data were summarised by the CTC and reviewed throughout the trial with the aim of identifying any issues with the recruitment. Patients reported as recruited, declined or not approached were classified as eligible. Approach rate was calculated as the proportion of those eligible who were approached (recruited + declined). Acceptance rates were calculated as the proportion of patients recruited of those approached. Free-text responses were categorised according to the standardised trial operating procedures.

Aggregate data were reviewed by the Trial Management Group (TMG) which provides ongoing day-to-day oversight of the trial, comprising the chief investigator and key co-investigators from a cross-section of participating sites. TMG review took place every 6 months, and screening data were used to inform revisions to the trial documentation as deemed appropriate and to estimate the impact of any such changes. Screening data were also regularly reviewed by the qualitative recruitment researcher.

In this retrospective exploratory analysis conducted by the CTC, groups were compared using Mann-Whitney *U* and, for proportions, Fisher’s exact test. Correlations were assessed using Pearson’s correlation coefficient (*r*). Data were analysed using Microsoft Excel and Stata v15.

## Results

**Fig. 3:**
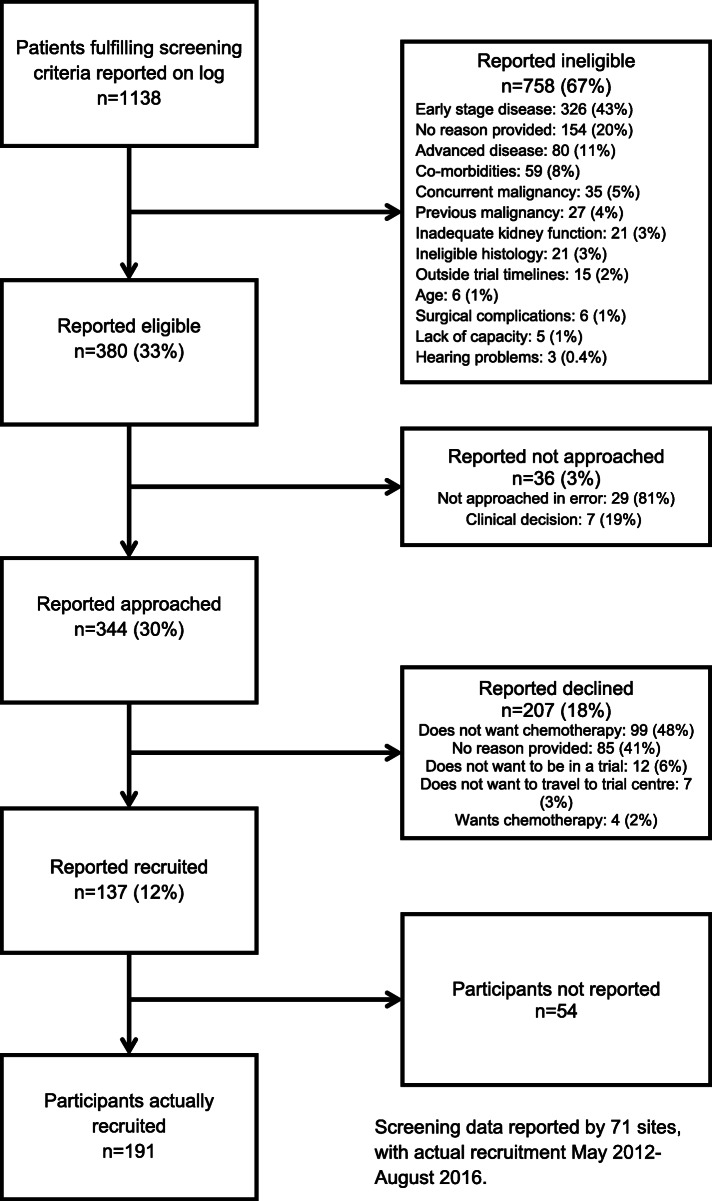
Screening data summary

The POUT trial opened to recruitment in May 2012. Detailed screening data were collected to August 2016. Seventy-one recruiting sites were open, representing 2768 centre months of recruitment activity in total during which 1138 patients were reported on screening logs and 191 participants joined the trial (Fig. 3).

Emails requesting logs were sent in 43/50 months between June 2012 and August 2016, a total of 2300 requests out of a possible 2762. The return rate following requests was 56% (1293 logs returned in response to 2300 requests), with a total of 18 logs submitted in the months when no request was sent (18/462 expected, 4% return rate). Overall, 1311/2762 expected monthly logs were received (47%). Seven centres (open for 71 centre months in total) did not return any logs.

Of the 191 patients actually recruited to August 2016, 54 (28%), recruited at 30 sites, were not reported on logs. Despite the ongoing CTC process of review and data cleaning in liaison with individual sites, including reminders to add all recruited patients to their logs, under-reporting of participants occurred throughout the trial with rates increasing towards the end of the screening period (Table 1).

**Table 1:** Under-reporting of recruited participants

Year	Total reported recruited	Actual recruitment	Total under-reported	Total centre months	Under-reporting per centre month
**2012 (May to December)**	11	10	− 1	145	− 0.007
**2013**	34	40	6	590	0.010
**2014**	41	48	7	759	0.009
**2015**	40	49	9	783	0.011
**2016 (January to July)**	11	44	33	491	0.067
**Total**	**137**	**191**	**54**	**2768**	**0.020**

Of the eight sites reporting no patients fitting the screening criteria throughout the duration of the screening period, three recruited one participant each. Six of the 25 sites which reported some screening data but no recruited patients had actually recruited participants (seven patients recruited across six sites).

Of the 1138 patients reported on the logs, 380 were categorised as eligible and 344 had been approached about participation (91% approach rate); 137 were reported as joining the trial—a 40% acceptance rate (207 declining participation).

The median monthly number of approached patients reported per centre was greater when a screening log request had been sent at the end of the month than when no request had been sent (median, 0.138 vs 0.062 patients/centre; Mann-Whitney *p* = 0.04) (Table 2). However, sending a request did not increase the median number of patients actually recruited the month after the request was sent, when we would expect such patients to join the trial according to protocol timelines (median, 0.062 vs 0.070 patients/centre; Mann-Whitney *p* = 0.39).

**Table 2:** Screening and recruitment numbers (all sites combined) by whether or not a screening log request was made

	Months	Total centre months logs expected	Total patients reported as approached	Median patients reported as approached/centre months	Total actual recruited next month	Median actual recruitment /centre month
**Screening log request sent**	43	2300	305	0.138	154	0.062
**Screening log request not sent**	7	462	39	0.062	39	0.070
**Total**	**50**	**2762**	**344**	**0.137**	**193**	**0.063**

**Fig. 4:**
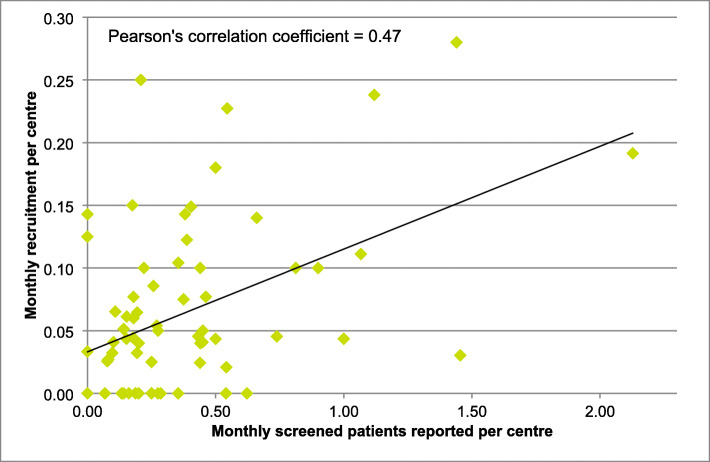
Overall recruitment rate per site vs patients reported as screened

A moderately strong positive correlation of 0.47 was observed between the mean monthly number of patients reported on screening and the mean number of participants each site actually recruited per month (Fig. 4). Sites recruiting more than the overall median of 0.04 patients per screening month reported a median of 0.38 screened patients per month, vs 0.20 for the lower recruiters (Mann Whitney *p* = 0.015). Higher-recruiting sites also reported a higher acceptance rate (*p* < 0.0001) (Table 3).

**Table 3:** Acceptance rates and screening activity by recruitment activity

	No. of sites	No. of centre months	Median pts reported monthly	Median eligible pts reported monthly	Median reported monthly recruitment	Median acceptance rate (%)	Median expected logs received (%)
Low-recruiting sites (under 0.04 patients per screening month)	35	1299	0.20	0.05	0.00	0	44.7
High-recruiting sites (over 0.04 patients per screening month)	36	1469	0.38	0.14	0.08	50.0	48.0
**Total**	**71**	**2768**	**0.26**	**0.10**	**0.03**	**33.3**	**47.1**

Sites were also categorised by whether they returned greater than the median 47% of expected logs to the CTC (high-returning sites) or not (low-returning sites). Whilst numbers reported as screened and eligible per month were greater for sites with higher numbers of screening log returns (*p* = 0.001 and *p* = 0.04), actual monthly recruitment was similar (*p* = 0.66) (Table 4.)

**Table 4:** Acceptance rates, screening and recruitment activity by compliance with returning screening logs

	No. of sites	No. of centre months	Median pts reported monthly	Median eligible pts reported monthly	Median reported monthly recruitment	Median acceptance rate (%)	Median monthly recruitment (actual)
Low-returning sites (under 47%)	35	1250	0.19	0.06	0.02	20.0	0.04
High-returning sites (over 47%)	36	1518	0.38	0.11	0.04	37.5	0.05
Total	71	2768	0.26	0.10	0.03	33.3	0.04

**Fig. 5:**
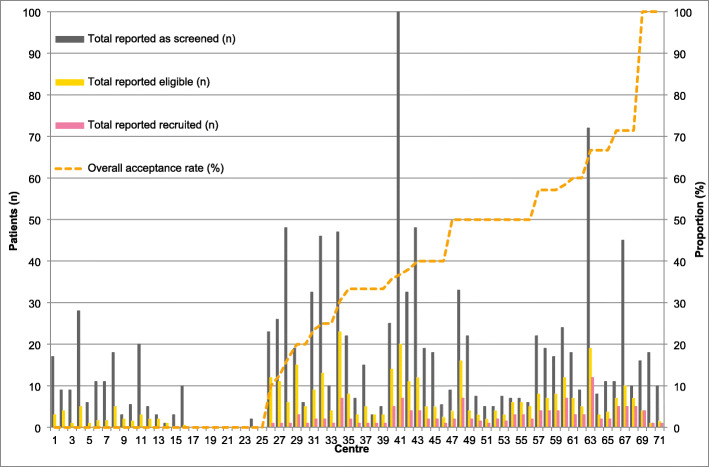
Screening data reported and actual recruitment per site by overall acceptance rate

There was considerable variability in screening activity per site, with no clear relationship between screening or recruitment activity and overall acceptance rate (Fig. 5).

The pre-surgery short patient information sheet did not appear to have any effect on acceptance rates. Of the 71 eligible patients who received the pre-surgery information sheet, 24 joined the trial (34%), whilst the acceptance rate amongst the 273 patients who did not receive a pre-surgery sheet was 41% (113/273) (*p* value, 0.277). The principal reason reported for declining was not wanting to receive chemotherapy, 99/207 (48%). Very few patients (4/207, 2%) declined due to preference for chemotherapy. In light of this observation, seen from the outset of the trial and supported by findings from the parallel qualitative study [22], the patient information sheet was reviewed and revised in 2014, with the aim of ensuring the information regarding the potential benefits and drawbacks of both surveillance and chemotherapy were outlined more clearly. From a retrospective review of reported screening data, the revisions to the patient information sheet made little discernible difference to the proportion of decliners who preferred not to have chemotherapy (48% with both versions); however, the overall acceptance rate did marginally increase following the implementation of the revised information sheet (38 to 42%) (Table 5).

**Table 5:** Impact of revisions to patient information sheet (PIS)

	Centre months	Patients approached (***n***)	Randomised (***n***)	Declined (***n***)	Overall acceptance rate (%)	Reason for declining: does not want chemotherapy (***n***)	Overall decliners due to not wanting chemotherapy (%)
**Previous version of PIS** (May 2012 to April 2014)	984	162	61	101	38	48	48
**Revised PIS** (May 2014 to July 2016)	1784	182	76	106	42	51	48
**Total**	2768	344	137	207	40	99	48

**Fig. 6:**
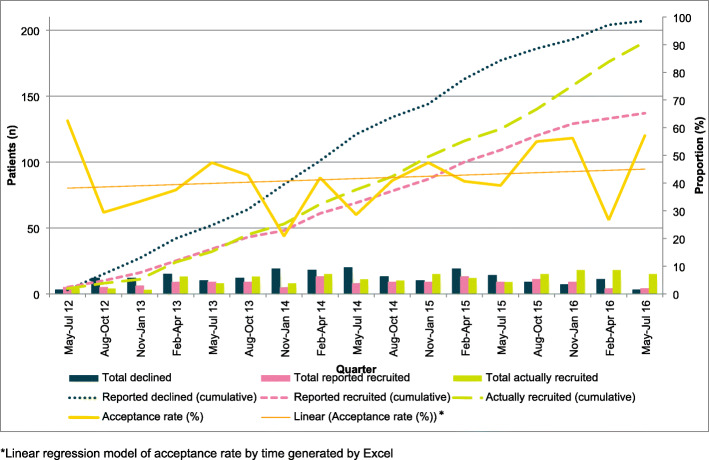
Screening patterns over time

**Fig. 7:**
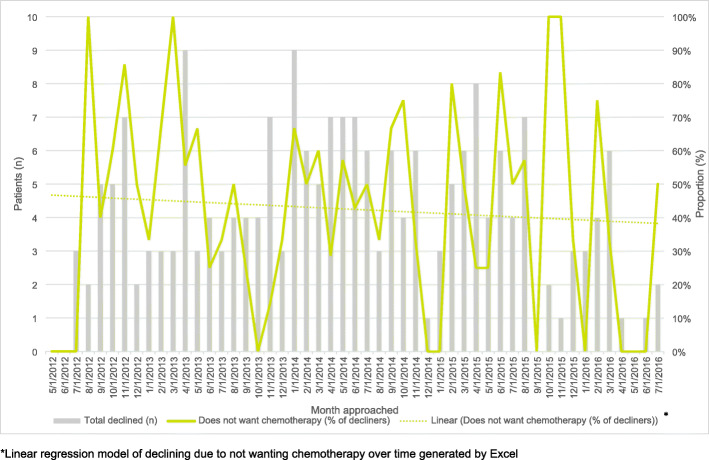
Decliners due to not wanting chemotherapy over time

Overall trends in recruitment rates remained relatively stable over time (Figs. 6 and 7).

## Discussion

Screening logs were a useful tool within the POUT trial as they demonstrated that the most common reason for patients declining participation was a preference not to undergo further treatment following surgery, contrary to the investigators’ initial expectations that patients would be keen to receive chemotherapy [22]. In the absence of the qualitative recruitment study, if screening logs had not been used, the investigators would have had a fundamental misunderstanding about why the majority of patients declined. In addition, the investigators’ expectation that providing information earlier in the patient pathway would improve trial acceptance was not confirmed: there was no indication that those patients approached prior to surgery demonstrated higher acceptance rates than those approached afterwards.

The proportion of eligible patients declining to participate was within the wide range of decline rates reported by other studies (15 to 80%) [15, 17, 23–26]. Whilst a preference against chemotherapy was not anticipated by the POUT investigators, declining due to treatment preference is consistent with the findings of a systematic review of participation in oncology trials [27]. Previous studies reporting screening and recruitment data do not provide details of the reasons for declining, instead presenting aggregated data or none at all [15, 18, 23, 24, 28]. Approach rates were good throughout the trial; however, centres may have been disinclined or unable to report patients who were missed, so the number not approached may have been under-reported.

We have demonstrated that screening logs were helpful in identifying a major reason for patients declining participation in the POUT trial; however, the attempt to redress this by amending the patient information sheet did not appear to have a major impact. Overall acceptance rates as reported on screening logs showed little variation throughout the trial. It is possible that prospective use of screening data to assess the impact of changes to essential documents or other recruitment interventions may have been more effective than the retrospective review conducted here [24].

The use of screening logs allowed near real-time central oversight of recruitment activity at those sites which complied with screening data reporting, enabling the CTC to identify and feedback any misinterpretations of eligibility criteria throughout the trial. Unfortunately, such interventions were not systematically recorded by the CTC; therefore, the impact of this feedback cannot be reported.

Screening data can also be used to inform revisions to eligibility criteria if patients in the target population are inadvertently excluded; however, no such changes were made within the POUT trial, despite the large proportion of ineligible patients reported. We suggest any substantial alteration of criteria should be approached with caution to avoid making the interpretation of results challenging or risk invalidating them entirely.

Whilst the CTC not sending a request for logs did reduce the number of patients reported for any given month, this did not appear to impact on the number of patients recruited in the subsequent month, suggesting that keeping and submitting screening logs may not in itself be sufficient to improve or support recruitment at sites.

Despite consistent requests for screening logs and querying of submitted data, under-reporting continued to be observed. Although this could only be confirmed by the CTC in the discrepancy between the number of patients reported as recruited and those actually randomised, it is likely that this under-reporting occurred across all categories. Whilst CONSORT recommends inclusion of screening data in clinical trial reports [20], our experience suggests that it is challenging and time-consuming to collect robust data from all participating sites, something that we did not succeed at despite our best efforts. Hence, the data provided to adhere to CONSORT recommendations may not be fully representative of all screening activity. The potential for bias should also be considered. It is feasible that those sites which routinely return screening data are unrepresentative of those which do not maintain or submit screening logs. Whilst we observed a correlation between the number of patients reported on screening and those recruited, as seen by previous investigators [19], this is likely to be confounded by the size of the patient population available for screening (and thus recruitment) at each site and so may not be an appropriate metric by which to measure trial activity and engagement. No correlation was observed between the proportion of expected logs submitted to the CTC by individual sites and their recruitment activity, which is inconsistent with previous findings [18].

The resource required to obtain robust screening data should not be underestimated. In our study, over 900 emails requesting logs received no response. Under-reporting of recruited patients persisted throughout the screening period, with the lower rate at the beginning reflecting protracted data cleaning efforts by the CTC and sites.

Maintenance and analysis of screening logs are time-consuming, and whilst they are intended to be a beneficial tool for sites, it may not be appropriate to implement them for all trials. There is a debate on their utility within the literature [18, 19], with little discussion of data quality and completeness in cases where they have been used [15, 17, 24]. If a trial has a large eligible patient population and recruitment is on target, the necessary workload may not be beneficial, either centrally or at site. In some studies which have reported screening data, the acceptance rate has been extremely low—for example, a prostate cancer trial reported 13,022 eligible men on screening, yet only 731 men were randomised [23]. This pattern has been observed in other studies [18, 29]. There is a risk that the workload associated with maintaining screening logs may disincentivise clinicians from trial participation [27, 30], especially if large numbers of ineligible patients have to be reported, so screening criteria should be carefully considered if logs are used.

In the POUT trial, the anticipated eligible patient population was small, so the workload for centres was not anticipated to be onerous. The main time requirement was at the CTC, principally as a result of the relatively large number of centres and the iterative process required to obtain clean data. On reflection, given that the approach and acceptance rates and the reasons for declining remained relatively stable throughout data collection, logs could have potentially been collected for a shorter period.

The inconsistency in definitions and reporting of screening data across studies makes it challenging to generalise findings across trials. If the SEAR framework, which proposes a similar data collection format as used here, is adopted, this should help standardise reporting of screening data [21]. It is possible that the utility of screening data varies between disease areas; however, standardised reporting of such data would help elucidate this. Across all settings, the cost-benefit ratio of collecting screening information should be considered, as it remains unclear whether routine use of logs substantially contributes to trial oversight or improves recruitment rates. We intend to investigate this further by embedding a study within future randomised controlled trials to prospectively investigate the utility of screening logs, the associated resource requirements and any impact they have on trial oversight and recruitment rates.

## Conclusions

Screening logs provided insight into reasons for non-participation within the POUT trial. Data reported remained consistent throughout the trial's duration, and no evidence was found that central collection of screening logs improved recruitment rates. The use of screening logs may not be appropriate for all trials or for the full duration of any given trial, and the resource requirements, both centrally and at site, should be carefully considered prior to their implementation. Despite their relatively widespread use, there exists a lack of evidence on the utility of screening logs in supporting or improving recruitment and this warrants further investigation within prospective studies.

## Data Availability

The datasets used during the current study are available from the corresponding author on reasonable request.

## Abbreviations

CONSORT: Consolidated Standards of Reporting Trials
CTC: Clinical trial coordinating centre
PIS: Patient information sheet
POUT: A phase III randomised trial of Peri-Operative chemotherapy versus sUrveillance in upper Tract urothelial cancer
SEAR: Screened, Eligible, Approached, Randomised
SPARE: Randomised trial of Selective bladder Preservation Against Radical Excision (cystectomy) in muscle invasive T2/T3 transitional cell carcinoma of the bladder: a feasibility study
TMG: Trial Management Group
UK: United Kingdom
UTUC: Upper tract urothelial carcinoma

## References

[1] Birtle A, Johnson M, Chester J, Jones R, Dolling D, Bryan RT (2020). Adjuvant chemotherapy in upper tract urothelial carcinoma (the POUT trial): a phase 3, open-label, randomised controlled trial. Lancet.

[2] Schott AF, Welch JJ, Verschraegen CF, Kurzrock R (2015). The national clinical trials network: conducting successful clinical trials of new therapies for rare cancers. Semin Oncol.

[3] Soria F, Shariat SF, Lerner SP, Fritsche HM, Rink M, Kassouf W (2017). Epidemiology, diagnosis, preoperative evaluation and prognostic assessment of upper-tract urothelial carcinoma (UTUC). World J Urol.

[4] Margulis V, Shariat SF, Matin SF, Kamat AM, Zigeuner R, Kikuchi E (2009). Outcomes of radical nephroureterectomy: a series from the upper tract urothelial carcinoma collaboration. Cancer.

[5] Rouprêt M, Babjuk M, Compérat E, Zigeuner R, Sylvester RJ, Burger M (2015). European Association of Urology Guidelines on Upper Urinary Tract urothelial Cell Carcinoma: 2015 update. Eur Urol.

[6] Bower P, Brueton V, Gamble C, Treweek S, Smith CT, Young B (2014). Interventions to improve recruitment and retention in clinical trials: a survey and workshop to assess current practice and future priorities. Trials.

[7] Tudur Smith C, Hickey H, Clarke M, Blazeby J, Williamson PR (2014). The trials methodological research agenda: results from a priority setting exercise. Trials.

[8] Huddart RA, Hall E, Lewis R, Birtle A (2010). Life and death of SPARE (Selective bladder Preservation Against Radical Excision): reflections on why the SPARE trial closed. BJU Int.

[9] Paramasivan S, Huddart R, Hall E, Lewis R, Birtle A, Donovan JL (2011). Key issues in recruitment to randomised controlled trials with very different interventions: a qualitative investigation of recruitment to the SPARE trial (CRUK/07/011). Trials.

[10] Mills EJ, Seely D, Rachlis B, Griffith L, Wu P, Wilson K (2006). Barriers to participation in clinical trials of cancer: a meta-analysis and systematic review of patient-reported factors. Lancet Oncol.

[11] Caldwell P, Hamilton S, Tan A, Craig J (2010). Strategies for increasing recruitment to randomized controlled trials: systematic review. PLoS Med.

[12] Donovan JL, Rooshenas L, Jepson M, Elliott D, Wade J, Avery K (2016). Optimising recruitment and informed consent in randomised controlled trials: the development and implementation of the Quintet Recruitment Intervention (QRI). Trials.

[13] Huddart RA, Birtle A, Maynard L, Beresford M, Blazeby J, Donovan J (2017). Clinical and patient-reported outcomes of SPARE - a randomised feasibility study of selective bladder preservation versus radical cystectomy. BJU Int.

[14] Nicola Mills MC, Young B, Murray G, Williamson P, Donovan J, Bhopal R, Jane Blazeby- on behalf of the Working Group. HTMR network top-tips for trial recruitment: HTMR Recruitment Working Group; 2013. https://www.methodologyhubs.mrc.ac.uk/files/5114/3403/2146/Recruitment_July2013V2.pdf.

[15] Kumar N, Crocker T, Smith T, Pow-Sang J, Spiess PE, Egan K (2012). Challenges and potential solutions to meeting accrual goals in a phase II chemoprevention trial for prostate cancer. Contemp Clin Trials.

[16] Mahajan P, Kulkarni A, Narayanswamy S, Dalal J, Halbe V, Patkar S (2015). Reasons why patients fail screening in Indian breast cancer trials. Perspect Clin Res.

[17] Sinclair H, Batty JA, Qiu W, Kunadian V (2016). Engaging older patients in cardiovascular research: observational analysis of the ICON-1 study. Open Heart.

[18] Slieker FJ, Kompanje EJ, Murray GD, Ohman J, Stocchetti N, Teasdale SG (2008). Importance of screening logs in clinical trials for severe traumatic brain injury. Neurosurgery.

[19] Elm JJ, Palesch Y, Easton JD, Lindblad A, Barsan W, Silbergleit R (2014). Screen failure data in clinical trials: are screening logs worth it?. Clin Trials (London, England).

[20] Schulz KF, Altman DG, Moher D (2010). CONSORT 2010 statement: updated guidelines for reporting parallel group randomised trials. BMJ.

[21] Wilson C, Rooshenas L, Paramasivan S, Elliott D, Jepson M, Strong S (2018). Development of a framework to improve the process of recruitment to randomised controlled trials (RCTs): the SEAR (Screened, Eligible, Approached, Randomised) framework. Trials.

[22] Wilson C, Snape M, Lewis R, Hall E, Johnson M, A B (2014). Recruitment challenges for trials of rare cancers: lessons from the POUT trial for transitional cell cancer of the urinary system CRUK/11/027.

[23] Wilt TJ, Brawer MK, Barry MJ, Jones KM, Kwon Y, Gingrich JR (2009). The Prostate cancer Intervention Versus Observation Trial: VA/NCI/AHRQ Cooperative Studies Program #407 (PIVOT): design and baseline results of a randomized controlled trial comparing radical prostatectomy to watchful waiting for men with clinically localized prostate cancer. Contemp Clin Trials.

[24] Pike NA, Pemberton V, Allen K, Jacobs JP, Hsu DT, Lewis AB (2013). Challenges and successes of recruitment in the “angiotensin-converting enzyme inhibition in infants with single ventricle trial” of the Pediatric Heart Network. Cardiol Young.

[25] Jones R, Jones RO, McCowan C, Montgomery AA, Fahey T (2009). The external validity of published randomized controlled trials in primary care. BMC Fam Pract.

[26] Sundaresan P, Turner S, Kneebone A, Pearse M, Fraser-Browne C, Woo HH (2014). Do screening trial recruitment logs accurately reflect the eligibility criteria of a given clinical trial? Early lessons from the RAVES 0803 Trial. Clin Oncol (R Coll Radiol).

[27] Fayter D, McDaid C, Eastwood A (2007). A systematic review highlights threats to validity in studies of barriers to cancer trial participation. J Clin Epidemiol.

[28] Lemieux J, Forget G, Brochu O, Provencher L, Cantin G, Desbiens C (2014). Evaluation of eligibility and recruitment in breast cancer clinical trials. Breast.

[29] St Germain D, Denicoff AM, Dimond EP, Carrigan A, Enos RA, Gonzalez MM (2014). Use of the National Cancer Institute Community Cancer Centers Program screening and accrual log to address cancer clinical trial accrual. J Oncol Pract.

[30] Rendell JM, Merritt RK, Geddes J. Incentives and disincentives to participation by clinicians in randomised controlled trials. Cochrane Database Syst Rev. 2007;(2). 10.1002/14651858.MR000021.pub3. 10.1002/14651858.MR000021.pub3 PMC7437389 17443636

